# Visualizing the Distribution of Jujube Metabolites at Different Maturity Stages Using Matrix-Assisted Laser Desorption/Ionization Mass Spectrometry Imaging

**DOI:** 10.3390/foods12203795

**Published:** 2023-10-16

**Authors:** Dongye Lu, Yang Wu, Junmin Zhang, Yuanyong Qi, Yuping Zhang, Qinghua Pan

**Affiliations:** 1 Institute of Forestry and Pomology, Beijing Academy of Agriculture and Forestry Sciences, Beijing 100093, China; ludongye123@163.com (D.L.); aboluo1983331@163.com (Y.W.); bjlgsbgs@126.com (Y.Q.); 2 Key Laboratory of Biology and Genetic Improvement of Horticultural Crops (North China), Ministry of Agriculture and Rural Affairs, Beijing 100093, China; 3 Beijing Engineering Research Center for Deciduous Fruit Trees, Beijing 100093, China; 4 Key Laboratory of Urban Agriculture (North China), Ministry of Agriculture and Rural Affairs, Beijing 100093, China; 5 Beijing Forestry Workstation, Beijing Municipal Forestry and Parks Buteau, Beijing 100013, China; barnard74@126.com

**Keywords:** *Ziziphus jujuba*, mass spectrometry imaging, spatial distribution, fruit quality

## Abstract

Chinese jujube (also called Chinese date, *Ziziphus jujuba* Mill.) is an economically important tree in China and provides a rich source of sugars, vitamins, and bioactive components, all of which are indispensable and essential for the composition and participation in life processes of the human body. However, the location of these metabolites in jujube fruits has not been determined. This study applied matrix-assisted laser desorption/ionization mass spectrometry imaging (MALDI-MSI) to investigate the spatial distribution of sugars, organic acids, and other key components in jujube fruits at different developmental periods. Soluble sugars such as hexoses and sucrose/maltose significantly increase with fruit ripening, while organic acids show an overall trend of initially increasing and then decreasing. Procyanidins and rutin exhibit specific distributions in the fruit periphery and peel. These findings suggest that MALDI-MSI can be used to study the spatial distribution of nutritional components in jujube fruits, providing insights into the changes and spatial distribution of substances during jujube fruit development. This technique offers a scientific basis for jujube breeding, utilization, and production.

## 1. Introduction

Chinese jujube (*Ziziphus jujuba* Mill.) is a native economic tree species in China with a long history of cultivation [1]. The jujube industry plays a significant role in rural revitalization and has greatly contributed to the economic development of rural areas in China [2,3]. Jujubes are grown on about 2 million hectares throughout the country, with an annual production of more than 8 million tons, and the dried jujube industry has become the main source of income for about 20 million farmers in China [2]. In addition to China, jujubes have been spread to at least 40 countries, including the United States, South Korea, Iran, Israel, Italy, and Australia [2,4,5,6]. The jujube fruit has high nutritional and medicinal value, earning it the reputation of a tonic since ancient times. It is even said that “eating three jujubes a day makes you immortal”. Jujube is abundant in sugars, protein, vitamins, trace elements, adenosine cyclic phosphate, alkaloids, flavonoids, triterpenoids, and other antioxidant components that can slow the progression of aging, lower blood sugar levels, and inhibit cancer cell growth [1,3,7,8].

The quality of jujube fruit is determined by its external characteristics like shape, size, color, and intrinsic qualities (e.g., nutrients, flavor, and soluble solids). The main metabolites that contribute to flavor and nutrition during jujube fruit development are sugars, organic acids, and volatile aromatic substances. However, studies have shown that the metabolites in jujube fruit vary at different developmental stages. For example, fructose and glucose accumulate in the early developmental period (young fruit stage), and sucrose rapidly accumulates in the late developmental period (white ripening stage), while the content of fructose and glucose also increases compared to the previous period [9]. The organic acid content of “Junzao” fruit increases in the early stage of development and decreases during ripening, following a high–low trend [9]. Furthermore, the content of these metabolites differs among different jujube varieties. In a study conducted by Lu et al. [10], metabolome analysis of two jujube cultivars was performed to identify metabolites that determine the sugar and acid taste of jujube fruit during its development. However, the spatial distribution of these metabolites has not been reported yet. Determining the location of these metabolites would not only provide valuable information for fruit quality research and jujube exploitation but also assist in post-harvest storage, processing, and breeding improvement.

Spatial metabolomics is a branch of metabolomics that has been developed using mass spectrometry imaging (MSI) technologies. This technology enables the visualization of metabolite locations within various tissues and organs without the need for extraction, purification, separation, or labeling [11]. By breaking the bottleneck of spatial information loss in traditional metabolomic studies, spatial metabolomics allows three-dimensional analysis: qualitative, quantitative, and localization. Among the various MSI technologies, matrix-assisted laser desorption/ionization mass spectrometry imaging (MALD-MSI) is a powerful tool that has found wide application in the study of cancer-related biomarkers and drugs [12,13] in the medical field, as well as in observation of the distribution of fruit metabolites in the field of food science [14,15,16]. Wang et al. [14] utilized MALDI-TOF IMS to visualize differences in the distribution of citric acid, soluble sugars, and anthocyanins in strawberries at four different maturity stages. The results demonstrated that citric acid and sugar were uniformly distributed throughout strawberry fruits at all ripening stages. Similarly, Kentaro et al. [17] employed this technology to visualize the distribution of soluble carbohydrates in apples (*Malus domestica*) and confirmed the utility of MALDI-MSI for examining carbohydrate metabolism during apple fruit maturation.

In this study, we employed MALDI-MSI analysis to investigate the spatial distribution of major metabolites in jujube fruits. These included soluble sugars, organic acid fractions, ascorbic acid, procyanidins, and hormones within the fruit of the jujube cultivar “Jingzao39” at four different developmental stages. Our findings will provide further insights into the phytochemical changes occurring during jujube fruit ripening from a spatial perspective. Moreover, they underscore the immense potential of MALDI-MSI technology in the areas of jujube quality improvement, breeding, and post-harvest production.

## 2. Materials and Methods

### 2.1. Reagents

Ethanol, methanol, acetonitrile, 2,5-dihydroxybenzoic acid (DHB), 4-hydoxy-cyanocinnamic acid (CHCA), 9-aminoacridine (9-AA), carboxymethyl cellulose (CMC) sodium salt, potassium dihydrogen phosphate, phosphoric acid, and HPLC-grade water were procured from Sigma-Aldrich (Shanghai, China). The standards of analyte were also provided by Sigma-Aldrich (Shanghai, China). All reagents, solvents, and standards used in the present study were of analytical grade.

### 2.2. Plant Materials

**Figure 1:**
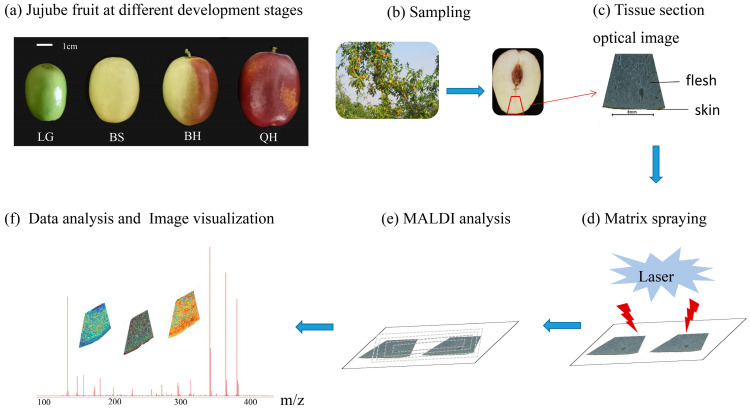
The matrix-assisted laser desorption/ionization MSI (MALDI-MSI) analysis of jujube fruits at different development stages: (**a**) jujube fruits at four stages (LG, BS, BH, and QH), and (**b**–**f**) experimental procedure of MALDI-MSI for analyzing jujube fruits.

“Jingzao 39” is a cultivar of fresh jujube characterized by its cylindrical shape, large size, and an average weight of 28.3 g. The “Jingzao 39” fruits used in this study were obtained from a nursery of germplasm resources at the Beijing Academy of Forestry and Pomology Sciences in Beijing, China. These jujube fruits were collected at four different ripening stages: green [LG, 50 days after anthesis (DAA)], white-ripened (BS, 80 DAA), half-red (BH, 100 DAA), and fully red (QH, 110 DAA) (Figure 1a). The collected fruits were immediately embedded in 1% carboxymethyl cellulose (CMC) sodium salt, frozen, and stored at −80 °C until further analysis.

### 2.3. Jujube Sample Preparation and Matrix Coating

For sample preparation and matrix coating, the frozen tissue samples were first fixed in three drops of distilled water during the cutting stage. The tissue samples were then sectioned at a thickness of 90 μm using a Leica CM1950 cryostat (Leica Microsystems GmbH, Wetzlar, Germany) at −20 °C. Due to the presence of nuclei and cavities and the larger fruit size of “Jingzao 39”, the lower part of each fruit was selected based on actual observations, and fan-shaped tissue sections were intercepted for subsequent analysis (Figure 1b). The typical process of MALDI-MSI is illustrated in Figure 1c–f. The tissue sections were arranged in groups on electrically conductive slides coated with indium tin oxide (ITO), and the slides with tissue sections were dried in a vacuum desiccator for 30 min.

The desiccated tissue sections mounted on ITO glass slides were then spray coated using an HTX TM sprayer (Bruker Daltonics, Bremen, Germany) with a solution of 15 mg/mL DHB (2,5-dihydroxybenzoic acid) dissolved in a mixture of 90% acetonitrile and 10% water. The sprayer temperature was set to 60 °C, with a flow rate of 0.12 mL/min and a pressure of 6 psi. Thirty passes of the matrix were applied to the slides, with a drying time of 5 s between each pass.

### 2.4. Mass Spectrometry Imaging

MALDI timsTOF MSI experiments were conducted using a prototype Bruker timsTOF flex MS system (Bruker Daltonics, Bremen, Germany) equipped with a 10 kHz smartbeam 3D laser. The laser power was initially set to 70% and remained fixed throughout the entire experiment. Mass spectra were acquired in the positive mode, covering a mass range from *m*/*z* 50 to 1300 Da. The imaging spatial resolution for the tissues was set to 50 μm, and each spectrum consisted of 400 laser shots. The tissue samples were detected under the same laser energy, and the laser beam was irradiated to the tissue area on the target plate through the grating. The samples were scanned continuously, with the tissue samples being ionized and resolved within the matrix under the excitation of the laser beam, and the released molecules were identified using the mass spectrometer to obtain the mass-to-charge ratio (*m*/*z*) information of each pixel of the samples, the peak raw data of each pixel of the samples, and the peak intensity.

### 2.5. Data Analysis

The raw data were imported into SCiLS Lab software v. 2022a (Bruker Daltonics, Bremen, Germany) for smoothing and root mean square (RMS) normalization to obtain the relative intensity information of different *m*/*z* at each spatial point, and the data were transformed into pixels on the imaging thermogram. The acquired MALDI mass spectra were normalized using the root mean square (RMS) method, and the signal intensity in each image was presented as the normalized intensity [18]. That is to say, using spatial resolution as a spot, RMS normalization was performed on the ion intensity in each spot, and the normalized intensity was used as the relative quantitative intensity information for subsequent data processing. For further detailed structural confirmation of the identified metabolites, MS/MS fragmentations were performed using the timsTOF flex MS system in the MS/MS mode.

The target peaks of the target substance (MS1 information) were subjected to on-tissue in situ fragmentation to obtain on-tissue MS/MS spectra (MS/MS fragment ion information). Substance identification was carried out by comparing the MS/MS spectra obtained with those of the reference standards in a self-built database MWDB (Metware Biotechnology Co., Ltd., Wuhan, China). For target peaks (MS1 information) with low intensity that could not be subjected to MS/MS fragmentation, substance identification at the MS1 level was performed based on the detected molecular weight. If the mass error range between the detected molecular weight and the theoretical monoisotopic molecular weight under the adduct mode of the target substance was within 10 ppm, the substance was identified at the MS1 level. A total of 18 substances were identified in this project, with all 18 substances identified at the MS/MS level. The distribution of soluble sugar metabolites on the tissue samples was obtained by imaging all the identified target peaks.

## 3. Results and Discussion

### 3.1. Matrix Selection

The selection of the matrix is crucial for the success of MALDI-MSI. Commonly used matrices for screening include DHB [19], CHCA [20], 9-AA [21], DMCA [22], and NEDC [23]. In this study, as shown in Table S1, it was found that using 1.5 mg/mL DHB detected the highest number of target metabolites in the positive ion mode, with 18 compounds, which is more than the 13 compounds detected using CHCA in the positive ion mode and the 10 species detected using 9-AA in the negative ion mode. Therefore, the DHB matrix was ultimately chosen for subsequent MALDI-MSI analysis in this study. Previous studies have also indicated that the DHB matrix is suitable for detecting sugars in plant tissues in the positive ion mode [24,25], while 9-AA is commonly used for analyzing organic acids and other compounds in the negative ion mode [26]. However, in this study, maltose, succinic acid, anthocyanins, and other substances were easily detected in the positive ion mode using the DHB matrix as well.

### 3.2. Identification of Metabolites in Jujube Samples

**Figure 2:**
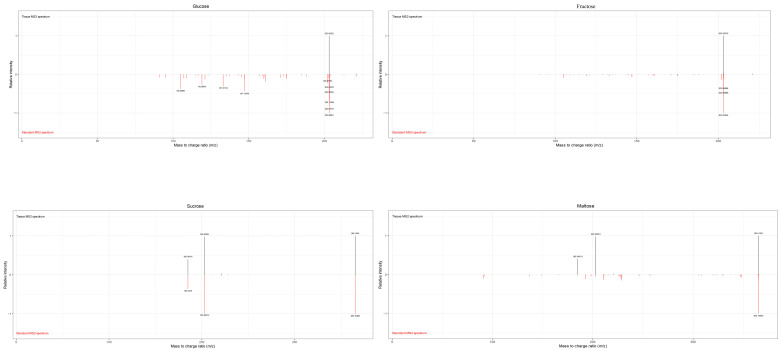
Comparison of secondary spectra of soluble sugars. Note: Below the horizontal axis (red) is the secondary spectrum of the target of the substance of interest, and above the horizontal axis (black) is the secondary spectrum of the substance collected on the tissue.

In this study, MALDI-MSI was performed to analyze the spatial distribution of soluble sugars, organic acids, plant hormones, vitamins, procyanidins, and flavonoid in jujube fruits at four different maturity stages (LG, BS, BH, and QH). The average mass MS spectra of the jujube tissues at different periods are shown in Figure S1. The relative ionic strength of sucrose and maltose (*m*/*z* 365.1053, isomer) increased significantly with an increase in fruit ripeness (Table 1). In the positive ion mode, by using the DHB matrix, succinic acid and quinic acid were detected as NH4^+^ adducts; 6-Benzylaminopurine and procyanidin A were detected as H^+^ adducts; and soluble sugars, partial organic acids (citric acid and isocitric acid), ascorbic acid, procyanidin B, and procyanidin C were detected as Na^+^ adducts (Table 1). In this study, for substances such as glucose, fructose, mannose, galactose (isomer) that could not be subjected to MS/MS fragmentation due to their low intensity, accurate identification could still be achieved based on a mass error range of 10 ppm between the detected molecular weight and the theoretical monoisotopic molecular weight under the adduct mode of the standard (Table 1). The identified substances including glucose, fructose, mannose, galactose (*m*/*z* 203.0529, isomer), sucrose, and maltose (*m*/*z* 365.1053, isomer) are shown in Figure 2. After spectral comparison with the standards, organic acids such as succinic acid (*m*/*z* 136.0622), citric acid and isocitric acid (*m*/*z* 215.0169, isomer), quinic acid (*m*/*z* 210.0890), ascorbic acid (*m*/*z* 199.0212); strong antioxidant substances including procyanidins A1, A2 (isomer, *m*/*z* 577.1345, isomer), B1, B2, B3 (isomer, *m*/*z* 199.0212), C1 (*m*/*z* 889.1980), and rutin (*m*/*z* 633.1424); plant hormones such as Indole-3-acetic acid (IAA, *m*/*z* 198.0534) and 6-Benzylaminopurine (6-BA, *m*/*z* 226.1054); and the secondary maps of these substances are shown in the Supplementary Figures S2, S3 and S4, respectively. The MS/MS spectra of other substances can also be found in the Supplementary Materials (Figures S2–S4).

### 3.3. Spatial Distribution of Soluble Sugars

**Figure 3:**
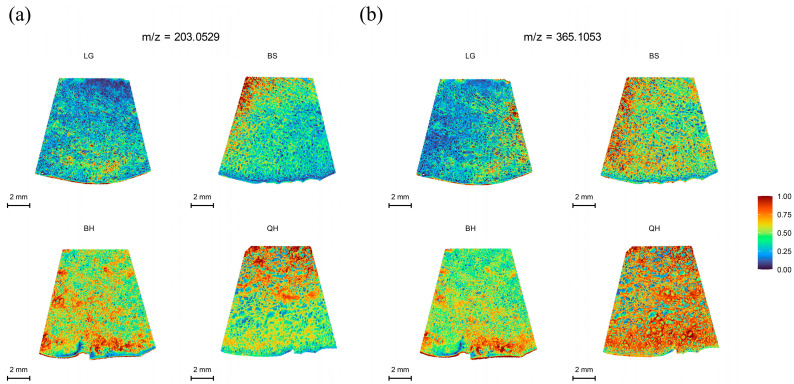
MALDI-MSI analyses of soluble sugars in jujube fruits at different development stages: (**a**) hexoses (glucose/fructose) at *m*/*z* 203.0529, and (**b**) sucrose/maltose at *m*/*z* 365.1053. Note: The lower left corner is the scale bar, and different colors represent different relative intensities of substances in the area. As shown in the legend, the content of the target substance increases sequentially from 0 to 1.

Sweetness is an important indicator that determines fruit quality and market value. Sugars such as glucose, fructose, and sucrose are the major sugars found in most fruits, including peaches, apples, watermelons, and cherries [27]. Each type of sugar represents a different level of sweetness [28], with fructose being the sweetest, followed by sucrose, and glucose being the least sweet. However, glucose exhibits the best fruit flavor [29]. These sugars play important regulatory roles in the growth, development, and ripening of fruits [30]. In practice, it is not possible to distinguish glucose from fructose using MALDI-TOF IMS alone because they have the same molecular weight (180.06284) [31]. The same situation occurs with sucrose and maltose, as they both have a molecular weight of 342.115663. Therefore, in this study, only the distribution of hexoses (glucose and fructose, *m*/*z* 203.0529) and sucrose/maltose (*m*/*z* 365.1053) in jujubes at four different developmental stages was determined. As shown in Figure 3, both hexoses and sucrose/maltose exhibited a significant increase from the LG stage to the QH stage, reaching the highest level at the QH stage (Figure 3a,b). These results are consistent with the findings of Zhao et al. [32] and Guo et al. [33] regarding soluble sugars in jujubes. Moreover, this trend is similar to the sugar accumulation trend observed in other fruits such as strawberries and wolfberries [14,34]. Previous studies have shown that sucrose acts as a signal for inducing fruit ripening and ABA accumulation, thereby participating in the regulation of fruit ripening [30,35]. This could explain why hexoses and sucrose/maltose show the highest intensity at the QH stage compared to other stages (Table S2).

### 3.4. Spatial Distribution of Organic Acids

**Figure 4:**
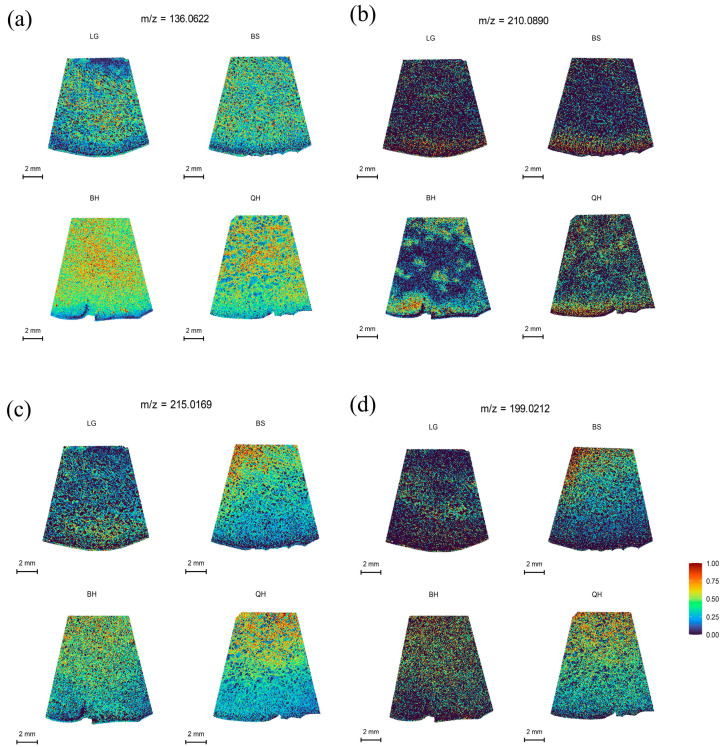
MALDI-MSI analyses of organic acids in jujube fruits at different development stages: (**a**) succinic acid at *m*/*z* 136.0622, (**b**) quinic acid at *m*/*z* 210.0890, (**c**) citric acid/isocitric acid at *m*/*z* 215.0169, and (**d**) ascorbic acid at *m*/*z* 199.0212.

Organic acids are important metabolic products in organisms that participate in various life activities, such as energy metabolism and other metabolic pathways. In plants, organic acids not only play a crucial role in the synthesis of primary metabolites, such as fatty acids and amino acids, but also serve as important factors determining the acidity, color, texture, and flavor of fruits [36,37]. The main organic acids in fruits include malic acid, citric acid, succinic acid, quinic acid, and ascorbic acid. Malic acid and citric acid are the most common organic acids found in fruits, such as peaches [38], strawberries [37], and jujubes [39]. It has been reported that jujubes are malic acid-accumulating fruits, with the highest content of malic acid, followed by quinic acid, citric acid, and succinic acid at the least amount. However, different varieties can have different types and amounts of organic acids. In this study, for example, except for malic acid, which was not detected under the DHB matrix, succinic acid at *m*/*z* 136.0622 was distributed most abundantly, followed by citric acid/isocitric acid at *m*/*z* 215.0169, while quinic acid at *m*/*z* 210.0890 was distributed the least (Table S2). Succinic acid showed a uniform distribution at all developmental stages and increased initially but decreased with fruit ripening (Figure 4a). Quinic acid exhibited a similar trend to succinic acid but was distributed in smaller amounts (Figure 4b). Citric acid was predominantly distributed in the pulp near the stone at all developmental stages and increased initially, then decreased, and increased again with fruit ripening (Figure 4c). However, some jujube cultivars may have higher levels of citric acid than succinic acid or no detectable presence of succinic acid [39].

Ascorbic acid, also known as vitamin C, is an important antioxidant that can prevent and inhibit cancer, protect against scurvy, lower cholesterol levels, and enhance immunity [3]. Jujubes are one of the fruits with the highest content of ascorbic acid, surpassing strawberries, apples, pears, peaches, and oranges (Liu et al. 2014), earning them the nickname “natural vitamin C pills.” It has been reported that in most jujube fruits, the content of ascorbic acid increases initially and then decreases during the ripening process [40,41]. In this study, as shown in Figure 4d and Table S2, the distribution of ascorbic acid at *m*/*z* 199.0212 in jujubes followed a trend of initially increasing, then decreasing, and increasing again from the LG stage to the QH stage, with higher levels of ascorbic acid found near the pulp than in the peel (Figure 4d).

### 3.5. Spatial Distribution of Procyanidins and Flavonoid

**Figure 5:**
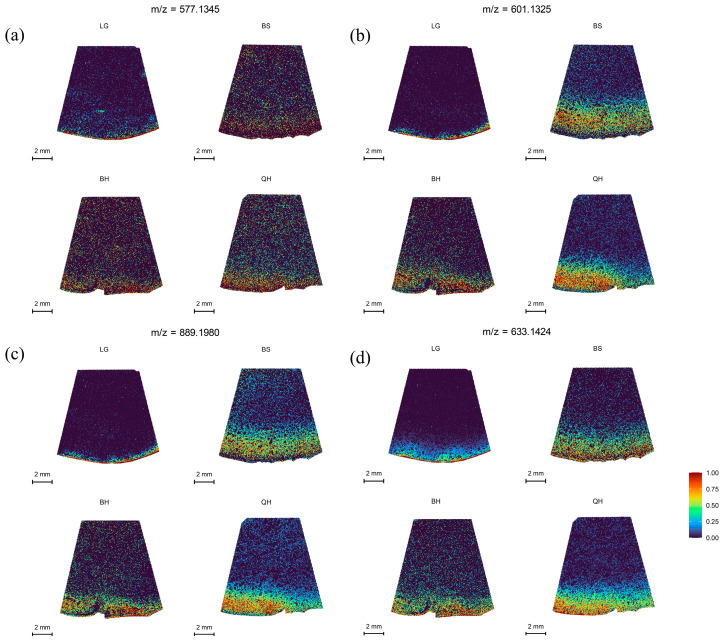
MALDI-MSI analyses of procyanidins and flavonoid in jujube fruits at different development stages: (**a**) procyanidins A1 and A2 (isomer) at *m*/*z* 577.1345, (**b**) procyanidin B1, B2, and B3 (isomer) at *m*/*z* 601.1325, (**c**) procyanidin C1 at *m*/*z* 889.1980, and (**d**) rutin at *m*/*z* 633.1424.

Procyanidins (PA), one of the major polyphenolic compounds in jujubes, possess various physicochemical properties and biological activities, including antioxidant, anticancer, and cardiovascular activities [42]. They contribute to the formation of pigments, flavors, and nutritional value [43]. In this study, the spatial distribution of procyanidin A at *m*/*z* 577.1345, procyanidin B at *m*/*z* 601.1325, and procyanidin C at *m*/*z* 889.1980 was analyzed, and the results are shown in Figure 5. Procyanidin A was found to be distributed in lower amounts in jujube fruits at different developmental stages (Figure 5a), and its relative intensity showed a decreasing trend followed by an increasing trend (Table S2). In contrast, procyanidin B and procyanidin C exhibited distinct distribution patterns in the fruit periphery and peel at different periods, showing an increasing trend followed by a slight decrease and then an increase again, with the highest distribution at the BS stage (Figure 5b,c). Rutin, a flavonoid compound, also contributes to fruit nutrition and pigment formation and has various beneficial health effects [44]. Rutin showed a characteristic of higher relative intensity at the LG and QH stages compared to the other two stages (Table S2), and its spatial distribution increased as the fruit ripened (Figure 5d). The higher distribution at the QH stage is associated with fruit ripening and the reddening of the fruit peel.

### 3.6. Spatial Distribution of Other Substances

**Figure 6:**
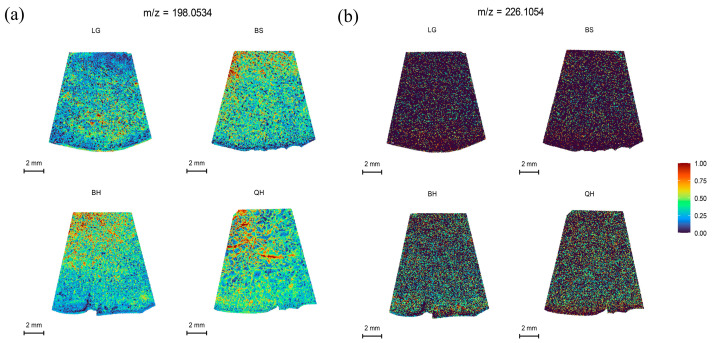
MALDI-MSI analyses of IAA and 6-BA hormones in jujube fruits at different development stages: (**a**) IAA at *m*/*z* 198.0534 and (**b**) 6-BA at *m*/*z* 226.1054.

Plant hormones are an indispensable part of regulating fruit development and ripening. Indole-3-acetic acid (IAA) and 6-benzylaminopurine (6-BA) belong to auxin and cytokinin, respectively. They are natural endogenous plant hormones that are associated with fruit development and ripening [45,46]. Indole-3-acetic acid has been shown to be involved in the initial signal of fertilization, regulating fruit size and maturity-related events by controlling cell division and cell expansion [46,47]. In this study, the spatial distribution of IAA at *m*/*z* 198.0534 and 6-BA at *m*/*z* 226.1054 during jujube fruit development was analyzed. The results showed that the relative intensity of IAA exhibited a decreasing trend followed by an increasing trend (Table S2). It was higher during the early rapid growth period of jujube fruits, which is consistent with the findings in cucumbers [47]. Compared to IAA, 6-BA had lower endogenous distribution and weaker ion intensity within jujube fruits (Figure 6b, Table S2). Therefore, in previous studies, 6-BA hormone or its combination with other hormones has often been used to enhance fruit setting and improve fruit quality through exogenous application to plants [48,49].

### 3.7. Prospects for MALDI-MSI Technology

MALDI-MSI technology is a mature imaging technique that has been used in research on food [14,16,17,34] and medicine [12,13,50]. In this study, this technology was utilized to analyze the dynamic distribution changes of important nutritional metabolites during the development of jujube fruits. The research results have significant implications for guiding production practices. Firstly, this study can enhance the utilization value of jujubes and guide breeding work. By understanding the distribution patterns and changes in soluble sugars, organic acids, and other traits related to jujube quality, it can guide the cultivation, harvesting, storage, and processing techniques, thereby improving fruit quality and edibility. Additionally, understanding the distribution of metabolites at different growth stages of jujubes can help identify metabolites related to jujube quality and yield, facilitating the selection of superior varieties and providing guidance and strategies for jujube breeding. Secondly, this study contributes to industrial development and utilization of jujubes. Understanding the preferred locations of compounds of interest (such as rutin and ascorbic acid) is beneficial for their extraction, which in turn aids in industrial development of jujubes and provides valuable insights for other fruits as well. Although MALDI-MSI technology is currently an effective technique, its application in the field is still limited, especially for small-scale farmers who may lack the knowledge and operational skills required for existing spectral imaging technologies. Therefore, it is recommended to prioritize the research, application, and upgrading of this technology in universities, research institutes, or companies. Future efforts should focus on promoting collaboration between academia, industry, and research institutions to guide fieldwork for farmers. Additionally, simplifying the sample preparation procedures and shortening the processing time should be the direction of technological advancements, provided that familiarity with sample structure is ensured. In conclusion, spatial metabolomics research holds immense potential in jujube breeding, fruit utilization, and fruit industry development, while also providing new perspectives and methods for related fields of research and application.

## 4. Conclusions

In this study, MALDI-MSI technology was used to identify and locate major metabolites, including soluble sugars, organic acids, procyanidins, rutin, and plant hormones, in jujube fruit sections at different developmental stages. The relative intensities and spatial distributions of these metabolites in jujube fruits at four different stages were compared. The results showed that soluble sugars such as hexoses and sucrose/maltose significantly increased with fruit ripening, while organic acids showed an overall trend of initially increasing and then decreasing. Procyanidins and rutin exhibited specific distributions in the fruit periphery and peel, which is related to their specific functions in the fruit. The findings of this study contribute to a better understanding of the distribution of various nutrients in jujube fruits at different developmental stages and provide a reliable basis for jujube breeding and fruit utilization.

## Figures and Tables

**Table 1:** The information of metabolites identified using MALDI-MSI in the positive ion mode.

Group	Compounds	Formula	Precursor (Da) *m*/*z*	Exact Precursor (Da) *m*/*z*	Adduct	Mass Error (ppm)
Soluble sugars	Glucose	C_6_H_12_O_6_	203.0529	203.052609	M+Na	1.43
	Fructose	C_6_H_12_O_6_	203.0529	203.052609	M+Na	1.43
	Sucrose	C_12_H_22_O_11_	365.1053	365.105432	M+Na	−3.62
	Maltose	C_12_H_22_O_11_	365.1053	365.105432	M+Na	−3.62
Organic acids	Succinic acid	C_4_H_6_O_4_	136.0622	136.060434	M+NH4	1.30
	Citric acid	C_6_H_8_O_7_	215.0169	215.016223	M+Na	3.15
	Isocitric acid	C_6_H_8_O_7_	215.0169	215.016223	M+Na	3.15
	Quinic acid	C_7_H_12_O_6_	210.0890	210.097214	M+NH4	−3.91
	Ascorbic acid	C_6_H_8_O_6_	199.0212	199.021309	M+Na	−0.55
Procyanidins and flavonoid	Procyanidin A1	C_30_H_24_O_12_	577.1345	577.134053	M+H	7.75
	Procyanidin A2	C_30_H_24_O_12_	577.1345	577.134053	M+H	7.75
	Procyanidin B1	C_30_H_26_O_12_	601.1325	601.131647	M+Na	1.42
	Procyanidin B2	C_30_H_26_O_12_	601.1325	601.131647	M+Na	1.42
	Procyanidin B3	C_30_H_26_O_12_	601.1325	601.131647	M+Na	1.42
	Procyanidin C1	C_45_H_38_O_18_	889.1980	889.195035	M+Na	3.33
	Rutin	C_27_H_30_O_16_	633.1424	633.142606	M+Na	−3.25
Plant hormones	Indole-3-acetic acid	C_10_H_9_NO_2_	198.0534	198.052549	M+Na	4.30
	6-Benzylaminopurine	C_12_H_11_N_5_	226.1054	226.108722	M+H	−1.47

## Data Availability

The data presented in this study are available from the corresponding author upon request.

## Supplementary Materials

The following supporting information can be downloaded at https://www.mdpi.com/article/10.3390/foods12203795/s1, Figure S1: The average mass spectra of imaging areas in jujube tissue samples at different periods. The *x*-axis represents the mass charge ratio, and the *y*-axis represents the average peak intensity value within this area after root mean square normalization; Figure S2: Secondary map of organic acids at different developmental stages of jujube fruit; Figure S3: Secondary map of procyanidins and flavonoid at different developmental stages of jujube fruit; Figure S4: Secondary map of plant hormones at different developmental stages of jujube fruit; Table S1: Matrix selection in this study; Table S2: Relative quantitative information of the substances in the test area at four development stages.
