# Supplementary figures and images for: Visualizing the Distribution of Jujube Metabolites at Different Maturity Stages Using Matrix-Assisted Laser Desorption/Ionization Mass Spectrometry Imaging
Source: Foods. 2023 Oct 16;12(20):3795. doi: 10.3390/foods12203795 (PMC10606910; doi:10.3390/foods12203795)

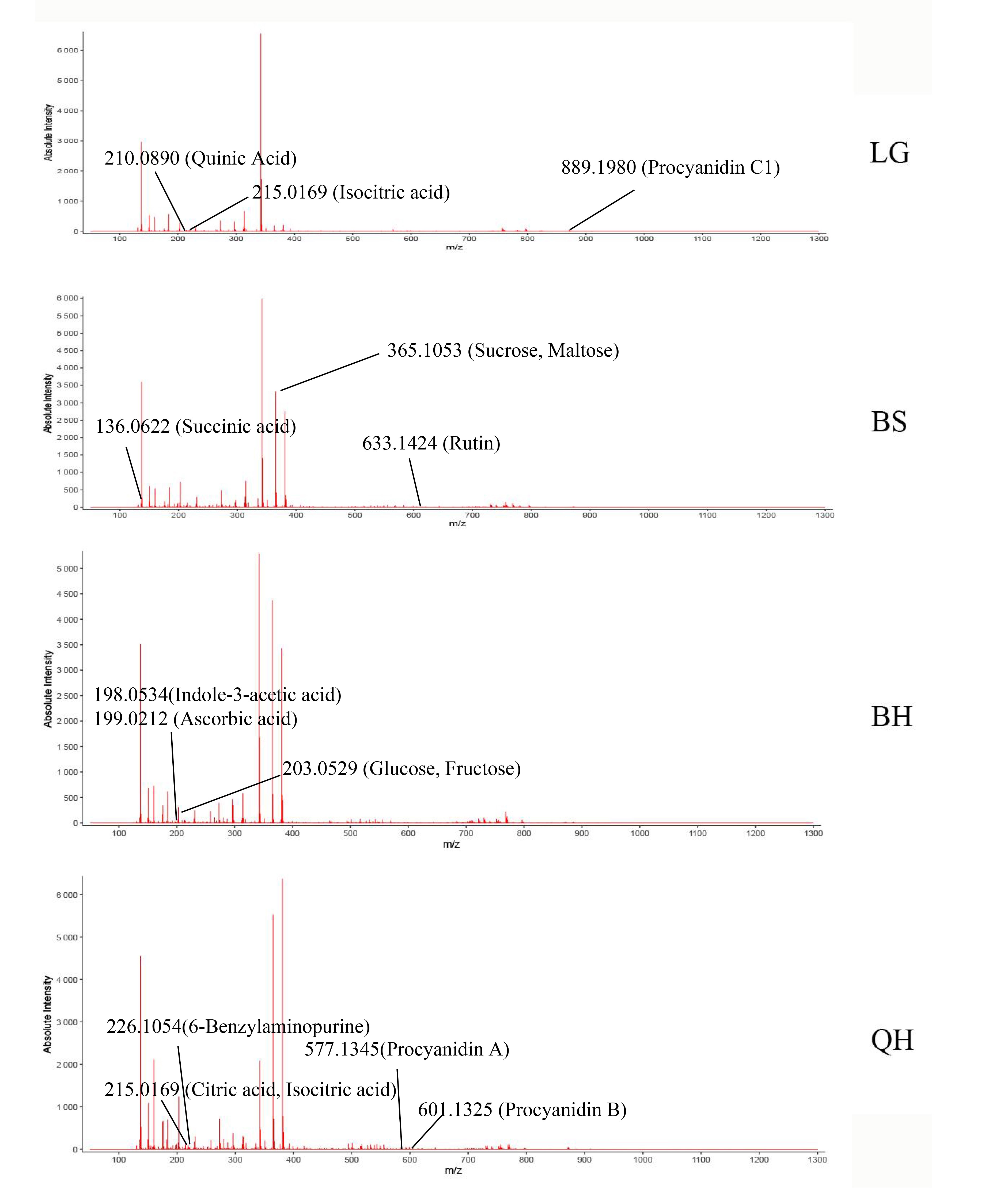

Supplement: Supplementary file 1 [file foods-12-03795-s001.zip › Figure S1.jpg]

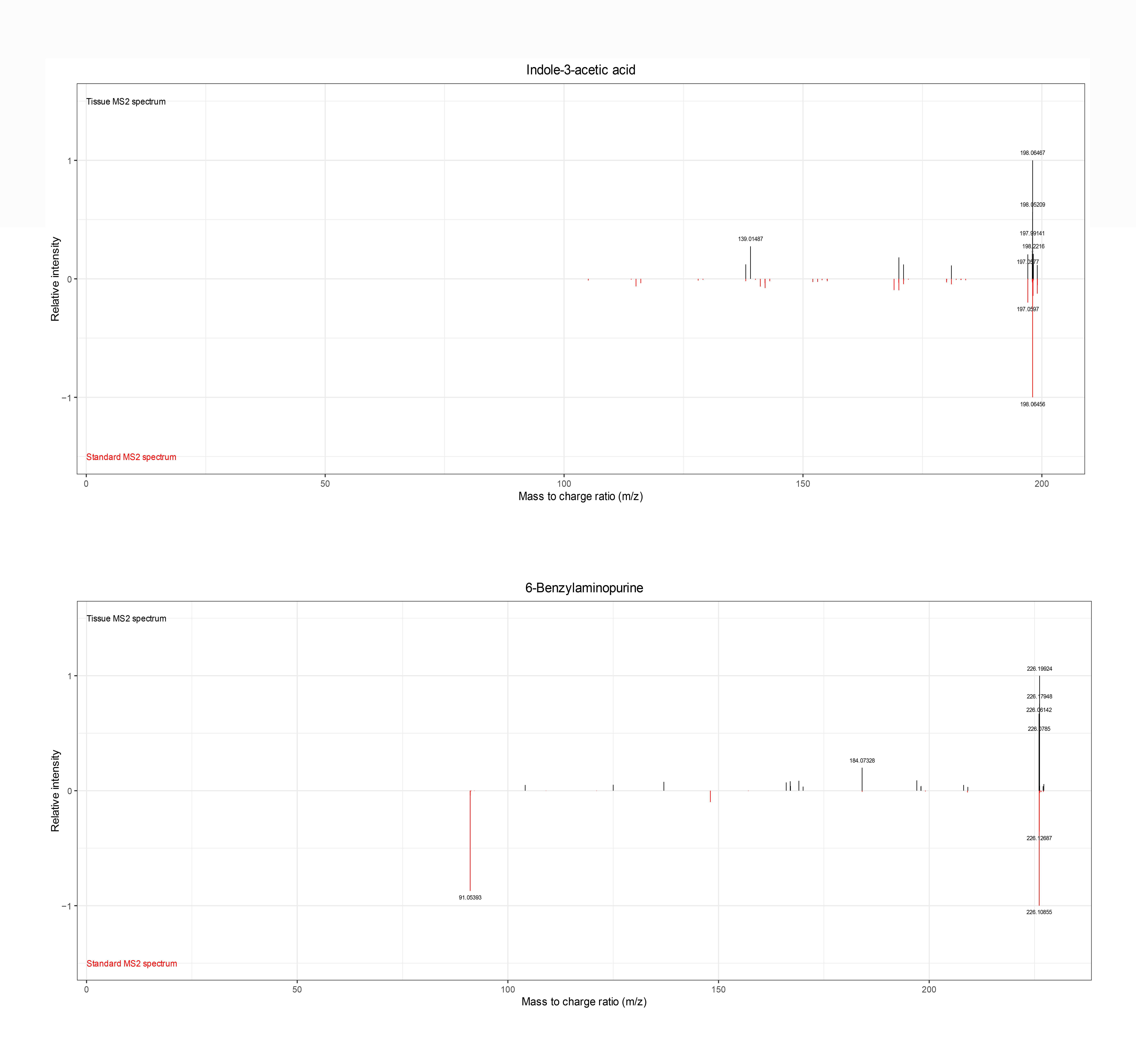

Supplement: Supplementary file 1 [file foods-12-03795-s001.zip › Figure S4.jpg]
